# Threshold analysis of rainfall and groundwater recharge in mitigating drought risks in overexploited groundwater regions

**DOI:** 10.1371/journal.pone.0341713

**Published:** 2026-02-04

**Authors:** Uneeb Ur Rehman Ali, Jinfeng Du, Muhammad Azram, Hassan Mujtaba Nawaz Saleem, Muhammad Hassan Raza

**Affiliations:** 1 School of Public Policy and Administration, Xi’an Jiaotong University, Xi’an, Shaanxi, China; 2 Institute of Business Management & Administrative Sciences, The Islamia University of Bahawalpur, Bahawalpur, Pakistan; 3 Human Settlements and Architectural Engineering, Xi’an Jiaotong University, Xi’an, Shaanxi, China; Escola de Engenharia de São Carlos da Universidade de São Paulo: Universidade de Sao Paulo Escola de Engenharia de Sao Carlos, BRAZIL

## Abstract

This study examines how rainfall and groundwater recharge can help mitigate drought conditions, using the Standardized Precipitation Evapotranspiration Index (SPEI) as the drought indicator. It focuses on the top ten countries experiencing groundwater overexploitation and incorporates a global average perspective to provide deeper insights into these critical relationships. These insights are essential for informed policy-making and integrated decision-making, involving a range of stakeholders from local users to international policymakers on drought mitigation efforts from 1961 to 2022. The analysis employs the novel technique to estimate Dynamic Panel Threshold Regression (DPThR) model. The findings reveal that a 1-millimeter increase in rainfall improves the SPEI by 0.003 units, thereby reducing drought likelihood. The threshold for mitigating drought effects is identified at 614.41 millimeters of annual rainfall, with Pakistan, Iran, and Saudi Arabia being the most at-risk countries when rainfall falls below this level. Conversely, a one-standard-deviation increase in groundwater recharge enhances the SPEI by 5.06 units, indicating a substantial reduction in drought incidence. The threshold for mitigating drought effects is identified at –0.0039 standard deviations, with China, Iran, Mexico, Pakistan, Saudi Arabia, Turkey, and the United States being the most drought-prone when recharge falls below this level. Furthermore, it was found that temperature exerts a consistently negative and highly significant effect, indicating that warming intensifies drought through evapotranspiration and soil moisture depletion. While CO_2_ emissions show no significant direct impact. Moreover, the study identifies unidirectional causality running from rainfall, groundwater recharge, temperature, and CO_2_ emissions, reinforcing the dominance of hydro-climatic forces in driving drought variability. Policy recommendations include advancing artificial rainfall, enhancing groundwater recharge, and maintaining country-specific water use thresholds to reduce drought risk and strengthen water and climate resilience in overexploited regions.

## 1. Introduction

Water scarcity and droughts are among the most pressing challenges facing the global community, with significant implications for ecosystems, economies, and human health. Climate change, combined with unsustainable water management practices, has exacerbated the severity and frequency of droughts, particularly in regions where groundwater is overexploited [1,2]. The top ten countries experiencing the highest levels of groundwater overexploitation, India, China, Pakistan, Iran, Indonesia, Bangladesh, Saudi Arabia, Turkey, the USA, and Mexico, are at the forefront of this crisis. According to the UN-Water [3], these regions are often facing severe consequences due to reduced water availability, highlighting the urgent need for effective drought mitigation strategies. Understanding how natural water cycles, such as rainfall and groundwater recharge, can mitigate the impacts of drought is essential for developing sustainable water management policies that ensure water security and climate resilience [4].

While the link between rainfall and drought has been widely studied, the role of groundwater recharge remains less understood. Existing research has often focused on causal relationships without addressing critical thresholds that determine when these factors are effective in mitigating drought risks. Moreover, much of the literature relies on linear models, which fail to account for the likely non-linear interactions between rainfall, groundwater recharge, and drought. The UNESCO [5] highlights the need to consider the growing pressures on groundwater resources and the importance of managing both groundwater and surface water for sustainable water use. To fill this gap, this study investigates the thresholds for rainfall and groundwater recharge that can mitigate drought in regions with significant groundwater overexploitation, using dynamic panel data models to capture the complexities of these relationships.

This study applies the recent advancement of Diallo [6] to estimate Dynamic Panel Threshold Regression (DPThR) model and the Juodis, Karavias [7] Panel Non-causality Test to analyze data from 1961 to 2022. This research is particularly timely given the growing recognition of the importance of groundwater recharge for drought resilience, yet empirical studies focusing on this relationship remain scarce. Furthermore, we explore the role of CO_2_ emissions and temperature changes, recognizing their influence on drought dynamics [8].

Unlike previous studies that have treated rainfall and groundwater recharge as linear, this research considers the non-linear nature of these variables’ impact on drought, using advanced econometric techniques to identify threshold effects. We also introduce the concept of unidirectional causality from groundwater recharge and rainfall to drought, challenging the traditional view that drought conditions primarily influence recharge rates. By applying multiple estimation methods, including several empirical equations for groundwater recharge, we ensure robust and reliable results.

This study makes the following key contributions to the field. First, it provides a comprehensive analysis of the rainfall and groundwater recharge thresholds required to mitigate drought in the world’s most overexploited groundwater regions. Second, it clarifies the direction of causality between drought and groundwater recharge, an area of ongoing debate in the literature. Finally, by incorporating advanced modeling techniques, this research offers a more nuanced understanding of the interactions between climate variables and drought, offering valuable insights for policy formulation.

However, the following are the specific objectives guide in the execution of the study: (*i*) to examine the nature and magnitude of the rate to which rainfall and groundwater recharge affects drought; (*ii*) to investigate the thresholds policy effect of rainfall and groundwater recharge capable of mitigating the effect of drought; (*iii*) to determine the countries at risk of drought with respect to the level of rainfall and groundwater recharge; and (*iv*) to undercover the theoretical argument about the specific direction of the causation of groundwater recharge in relation to drought.

The remainder of this paper is structured as follows: Section 2 reviews the relevant literature. Section 3 describes the data and methodology. Section 4 and 5 presents the results and discussion. Lastly, Section 6 concludes with policy recommendations.

## 2. Literature review

### 2.1. Rainfall and drought

The relationship between rainfall and drought has been extensively studied due to its significant implications for water resource management, agriculture, and socio-economic development. This theoretical framework aims to provide a comprehensive understanding of the complex interplay between rainfall patterns and drought occurrence, drawing on relevant literature and empirical evidence. Climate change is identified as a key driver of rainfall variability, affecting the frequency, intensity, and spatial distribution of precipitation [9]. As global temperatures rise, shifts in atmospheric circulation patterns and moisture availability can alter regional rainfall patterns [10]. These changes can result in prolonged dry spells and reduced overall precipitation, increasing the likelihood of droughts.

Drought is commonly defined as a prolonged period of abnormally low precipitation that leads to water scarcity and negatively impacts various sectors [11]. A rainfall deficit, the difference between actual and expected precipitation, serves as a crucial indicator of drought severity [12]. A sustained lack of rainfall can deplete soil moisture, reduce water availability in rivers and reservoirs, and hinder groundwater recharge, thus exacerbating drought conditions [4]. Moreover, the El Niño-Southern Oscillation (ENSO), a natural climate phenomenon marked by the periodic warming (El Niño) and cooling (La Niña) of the tropical Pacific Ocean, has been linked to drought occurrences. El Niño events often result in reduced rainfall in areas, like semi-arid basins in South Asia and the Middle East, while La Niña events can bring above-average precipitation [13].

Drought is a complex natural hazard that impacts ecosystems and society in many ways. It is generally classified into four categories: meteorological, agricultural, hydrological, and socioeconomic droughts. Meteorological drought refers to a precipitation deficiency, possibly combined with increased potential evapotranspiration, extending over a large area and spanning an extensive period of time. Agricultural drought is a lack of water damaging human agricultural activities, resulting from a combination of agricultural activities and natural phenomena, leading to insufficient water volumes or quality for plant and/or animal needs. Hydrological drought is related to negative anomalies in surface and subsurface water, manifesting as below-normal groundwater levels or water levels in lakes, declining wetland area, and decreased river discharge. Socioeconomic drought occurs when the demand for an economic good exceeds supply as a result of a weather-related shortfall in water supply [14].

Understanding the relationship between rainfall and drought is crucial for drought prediction and early warning systems. Reduced rainfall can also lower evapotranspiration rates, leading to drier soils and decreased moisture availability for subsequent rainfall events [15]. Furthermore, drought intensity refers to the magnitude of drought relative to its duration, while drought severity reflects the degree of rainfall deficit or the level of impact from the deficit [16]. Precipitation plays a vital role in influencing trends in drought occurrence, frequency, and duration within a region [17]. Therefore, analyzing precipitation and drought data is essential for developing effective water management strategies, environmental protection, agricultural planning, and understanding the broader economic impacts of drought [18–20].

Empirical studies found that, rainfall and drought are two natural phenomena that have significant impacts on various aspects of the environment, agriculture, and society. Understanding the relationship between rainfall and drought is crucial for effective water resource management and adaptation to climate change. In this review, we explore existing research on the relationship between rainfall and drought. One study by Sheffield, Wood [21] investigated the link between rainfall and drought in the Sahel region of Africa. The researchers found that below-average rainfall during the wet season was a primary driver of drought conditions in the region. They also emphasized the importance of considering both the quantity and distribution of rainfall when predicting drought events.

Another study by Dai [22] examined global drought patterns and their relationship with rainfall variability. The study found that regions with lower average rainfall were more susceptible to droughts, though the timing and intensity of rainfall events also played a significant role in drought occurrence. Dai highlighted the need for improved monitoring and forecasting of rainfall patterns to better understand and mitigate drought impacts. A study by Gutzler and Robbins [23] focused on rainfall variability and drought in the United States. They discovered that changes in rainfall patterns, such as increased variability and intensity, were linked to more frequent and severe droughts in certain regions.

### 2.2. Groundwater recharge and drought

The relationship between groundwater recharge and drought has been extensively studied in the literature. According to the United States Geological Survey (USGS), groundwater recharge refers to the process by which water enters the ground and replenishes the aquifer [24], while drought is characterized as a prolonged period of abnormally low rainfall leading to a water shortage [25]. One of the key strategies for mitigating the effects of drought is enhancing groundwater recharge, which involves replenishing depleted aquifers to improve water availability during dry periods. This process has gained significant attention in hydrology and environmental management, especially in regions prone to severe drought conditions.

Several theoretical frameworks help explain the critical role of groundwater recharge in maintaining hydrological stability and building resilience against drought. The first is the Water Balance Model, which provides a foundational approach to understanding recharge by assessing the relationship between precipitation, infiltration, evapotranspiration, and groundwater extraction [26].

Another relevant framework is the sustainable groundwater management theory, which underscores the importance of long-term groundwater conservation strategies to support ecological stability and socio-economic resilience [27]. This theory advocates for controlled artificial recharge techniques such as rainwater harvesting, managed aquifer recharge (MAR), and subsurface dam construction to enhance groundwater storage. Finally, the Hydrogeological Systems Approach examines groundwater recharge from a geophysical perspective, focusing on the interaction between soil permeability, aquifer properties, and climatic conditions [28]. This approach highlights that recharge rates are influenced by geological formations, aquifer depth, and precipitation variability, which necessitate localized strategies for effective water conservation. A thorough understanding of hydrogeological characteristics allows policymakers to design targeted interventions that are tailored to regional drought vulnerabilities.

From the existing empirical studies, McEvoy, Famiglietti [29] found that increased groundwater recharge in California helped mitigate the effects of drought. Similarly, Guo, Dirmeyer [30] investigated the effects of groundwater recharge on vegetation dynamics during a severe drought. They found that areas with higher recharge rates exhibited greater vegetation resilience, underscoring the importance of groundwater recharge for maintaining ecosystem health. However, some studies have shown that drought can significantly affect groundwater recharge, creating a bidirectional relationship between recharge and drought. For instance, Scanlon, Keese [31] found that drought conditions in the Texas High Plains led to a decrease in groundwater recharge. Similarly, Guo, Dirmeyer [30] observed that drought conditions in the Amazon basin resulted in a reduction in groundwater recharge.

### 2.3. Gaps in the literature

A review of the extant literature highlights the critical importance of understanding the complex interplay between rainfall and drought for the development of effective mitigation and adaptation strategies. Empirical studies exploring this dynamic relationship are vital, yet several gaps remain in the literature that need to be addressed to further refine our understanding and policy responses. Firstly, existing studies fail to provide a comprehensive understanding of the underlying mechanisms between rainfall and drought, and there is a notable lack of robust models for drought prediction and management. This deficiency limits the capacity for accurately forecasting drought events and formulating targeted interventions that can prevent or alleviate their impacts. The development of more advanced and reliable predictive models is essential for improving drought resilience. Secondly, while much research has focused on the relationship between groundwater recharge and drought, the intricacies of this connection remain insufficiently explored. Groundwater recharge and drought are interdependent in complex ways drought can diminish groundwater recharge, while an increase in recharge can mitigate the severity of drought. This cyclical interaction highlights the need for further investigation into the direction of causality between groundwater recharge and drought. Understanding this causal relationship is crucial for designing effective management strategies that address both groundwater depletion and drought mitigation. Thirdly, a significant portion of the existing literature predominantly focuses on causal inference, often neglecting practical issues such as the establishment of policy thresholds for artificial rainfall and groundwater recharge. Determining these thresholds is critical for developing actionable policies that can proactively manage water resources and mitigate drought risks. Without this foundational work, it remains difficult to craft policies that can effectively address local water needs in the face of varying climatic and hydrological conditions. Finally, while the relationships among rainfall, groundwater recharge, and drought may be linear or nonlinear, the literature does not consistently clarify the nature of these relationships, especially when employing advanced methodologies.

## 3. Methodology

### 3.1. Data sources

The primary drought variable is the Standardized Precipitation-Evapotranspiration Index (SPEI). A higher SPEI value indicates a wetter condition (less drought). Therefore, in our analysis, a positive correlation with another variable suggests that variable contributes to drought mitigation, while a negative correlation suggests it contributes to drought intensification.. Specifically, the SPEI is widely recognized for its ability to capture the combined effect of precipitation deficits and temperature variations, which makes it particularly suited for assessing drought intensity and frequency in the context of climate change and water resource management.

The SPEI is employed as the dependent variable due to its incorporation of precipitation and potential evapotranspiration, the latter of which exhibits a temperature-dependent response. The SPEI is a drought index that fulfills the aforementioned requirements due to its multi-scalar character, which enables its use by different scientific disciplines for the detection, monitoring, and analysis of droughts. The SPEI has been demonstrated to be a reliable metric for quantifying drought severity, as it incorporates both intensity and duration into its calculation. Furthermore, the SPEI has been shown to possess the capability to identify the onset and cessation of drought periods. The SPEI facilitates the comparison of drought severity across various temporal and geographical scales, given its capacity to be calculated over a broad spectrum of climates, a capability shared by the SPI [32]. The independent variables include rainfall (average precipitation in depth) (*Rainfall*); groundwater recharge (*GW*_r_); temperature (temperature change per year in ^0^C) (*Temperature*) to capture warming trends that alter potential evapotranspiration and thus SPEI; and CO_2_ emissions (CO_2_ emissions per capita metric tons) (*CO*_*2*_ *emissions*) as an indicator of anthropogenic radiative forcing and of socio-economic activity that correlates with land-use change and energy use, and that higher emissions can contribute to regional warming and evaporative demand and therefore alter drought risk.

However, this study makes a global sample of the top ten countries facing the problem of underground water overexploitation such as India, China, Pakistan, Iran, Indonesia, Bangladesh, Saudi Arabia, and Turkey which are all in the Asia-Pacific region. These eight countries collectively account for 75% of the total global groundwater withdrawal. However, the 2 remaining countries are the USA and Mexico which are both in the North America. This selection of the top 10 is according to the United Nations World Water Development Report [33]. The data range covers the period from 1961 to 2022, and the selection of the data range was based on data availability. The sources of the data include world development indicators [34], Climate Change Knowledge Portal [35], Global SPEI database [32], India Environment Portal [36], and Gapminder [37].

This study will calculate groundwater recharge using nine empirical equations or formulae. The following formulae have been developed by various researchers to calculate groundwater recharge. Nevertheless, the rationale behind employing multiple formulae, not merely a single one, lies in the nature of this study. It is a global sample, encompassing diverse regions worldwide. This approach acknowledges the absence of a universally applicable formula, underscoring the necessity for customized solutions tailored to the specific characteristics of each nation. In addition, the utilization of multiple methodologies is crucial for ensuring the precise impact of the groundwater recharge in relation to the outcome variable (drought), thereby ensuring accuracy of the actual impact. Furthermore, the utilization of a diverse array of methodologies is a prevalent practice in current empirical research, as evidenced in the works of Andualem, Demeke [38] and Addisie [39].

To calculate the level of groundwater recharge for each country with maximum efficiency, the nine different formulae were used to calculate the level of groundwater recharge for each country. Ultimately, an index of the nine different observations of the groundwater recharge for each country was calculated using Principal Component Analysis (PCA) after standardizing the observations from the different formulae. This was necessary because the observations are at different measurement scales as the formulae are at different measurement scales, i.e., inch, millimeter, and centimeter. In addition, standardizing the observations will eliminate the issue of the different scales of measurement by the formulae. This will result in the production of a single scale variable as an index. Furthermore, PCA standardizes these estimates of groundwater across nations using varying methodologies, thereby attenuating methodological bias. The nine formulae encompass the Chaturvedi; Modified Chaturvedi; Sehgal; Krishina; Kirchner; Bredenkamp; Bhattacharjee; Kumar; and Maxey and Eakin. The nine formulae presented in Table 1.

**Table 1:** Empirical equations selected to estimate the variable groundwater recharge.

S/No.	Formula name	Equation	Remarks	Developed by
1	Chaturvedi	R = 2.0*(P – 15)^0.4^	P(inch)	Chaturvedi [40]
2	Modified Chaturvedi	R = 1.35*(P – 14)^0.5^	P(inch)	Kumar and Seethapathi [41]
3	Sehgal	R = 2.5*(P – 0.6)^0.5^	P(inch)	Ali, Mubarak [42]
4	Krishina	R = 0.25*(P – 400)	600 < P < 1,000, P(mm)	PR [43]
5	Kirchner	R = 0.12*(P – 20)	P(mm)	Kirchner, Van Tonder [44]
6	Bredenkamp	R = 0.32*(MAP – 360)	P(MAP)	Bredenkamp, Botha [45]
7	Bhattacharjee	R = 3.47*(P – 38)^0.4^	P(cm)	Deshbhandari and Krishnaiah [46]
8	Kumar	R = 0.63*(P – 15.28)^0.76^	P(inch)	Kumar and Seethapathi [41]
9	Maxey and Eakin	R = P*a	P(mm), a = 20%	Maxey and Eakin [47]

Source: Authors compilation

Table 1 is the nine empirical equations, i.e., the formulae used in estimating the underground water. From the table, the groundwater recharge throughout the year is represented by the symbol *R*, which is expressed in inches (*inch*), millimeters (*mm*), centimeters (*cm*), and mean annual rainfall (*MAP*). The annual precipitation is denoted by the symbol *P*, which is measured in inches, millimeters, centimeters, and mean annual rainfall. The asterisk (*) is stands for multiplication.

Upon applying PCA to the observations obtained from the nine formulae for each country, it was found that the first component (ground water variable calculated using formula 1 in Table 2) explained a large portion of the variation, reaching approximately 97% of the total variance. This finding indicates a strong convergence among the formulae. Therefore, the PCA-based index was predominantly characterized by the initial formula.

**Table 2:** Descriptive statistics for the panel countries.

Variable	Obs.	Mean	Std. Dev.	Min	Max
Drought	620	−.091	1.146	−5.875	2.805
Rainfall	620	945.302	845.264	74.56	3275.81
Temperature	620	.449	.592	−.985	2.37
CO_2_ emissions	620	4.897	6.256	.046	23.1
GWr	620	0	1.865	−2.689	4.346

Source: Author’s computation

To ensure cross-country comparability, the PCA index was employed as the representative measure of groundwater availability. A compelling argument for the utilization of PCA over a single component is the recognition that certain countries or regions possess formulae that are more aptly aligned with their distinct hydrogeological characteristics, such as arid versus tropical conditions. The adoption of a uniform formula could potentially result in the loss of regional representativeness, underscoring the importance of employing PCA to ensure the capture of local hydrogeological nuances.

Consequently, the PCA-based composite index is preferable as it ensures cross-country comparability and mitigates potential bias from any single formula at the country level. The alpha value of the items was found to be 0.8920.

### 3.2. Functional specifications of the relationship

To circumvent the issue of multicollinearity, the study formulated two distinct model specifications. This approach was adopted to prevent multicollinearity, given that both groundwater recharge and rainfall serve as explanatory variables. The specifications were then implemented to analyze each variable independently, thereby ensuring the mitigation of multicollinearity and facilitating a nuanced examination of the impact on groundwater recharge. Therefore, the specifications expressed in equations (1) and (2) as follows:


$$Drought_{i,t}=\beta_{0}+\beta_{1}Rainfall_{i,t}+\beta_{2}Temperature_{i,t}+\beta_{3}CO_{2}emissions_{i,t}+e_{i,t}$$ (1)

$$\begin{array}{c} Drought_{i,t}=\beta_{0}+\beta_{1}Groundwaterrecharge_{i,t}+\beta_{2}Temperature_{i,t}\\ +\beta_{3}CO_{2}emissions_{i,t}+e_{i,t} \end{array}$$ (2)

where *Drought* is the response variable while *Rainfall, Groundwater recharge, Temperature,* and *CO*_*2*_ *emissions* are explanatory variables. β_0_ is the constant term; β_1_, β_2_, β_3_, and β_4_ are the coefficients of the respective explanatory variables, *e*_i,t_ is the stochastic term for country *i* at time *t*. However, in equation (1), the a priori expectations are as follows: β_1_ < 0, β_2_ > 0, and β_3_ > 0. While in equation (2), the a priori expectations are as follows: β_1_ <>0, β_2_ > 0, and β_3_ > 0.

### 3.3. Estimation techniques

Considering the study involves the estimation of causal impact and threshold level (policy threshold), the appropriate estimation technique to use is the recent advancement of Diallo [6] to estimate DPThR model for achieving the objectives of the study. This model accounts for possible asymmetric effects of exogenous variables based on whether the threshold variable is above or below an unknown threshold which allows for more nuanced analysis. Moreover, it is unlike static models, the dynamic panel threshold model considers lagged dependent variables and endogenous covariates. Furthermore, its asymptotic distribution is nonstandard as the number of individuals increases to infinity over a fixed time period, and it is consistent. Additionally, the slope estimates are asymptotically normally distributed and consistent. Again, the model addresses potential endogeneity issues by incorporating lagged variables and endogenous covariates, and this is crucial for accurate estimation in real-world applications. More so, considering dynamic features and threshold effects, this model provides a robust framework for empirical analysis. For the DPThR model, this study makes use of Panel Non-causality test by Juodis, Karavias [7] to provide the robustness check for the findings obtained from the DPThR model by checking whether there is consistency of the results from both methods.

#### 3.3.1. Dynamic Panel Threshold Regression (DPThR).

Following Seo, Kim [48], the estimation model of the study is in line with Dynamic Panel Threshold Regression (DPThR) model given in equation (3) as follows:


$$y_{i,t}=x{}^{\prime }{}_{i,t}\beta +(1,x{}_{it}^{\prime })\delta 1\{q_{i,t}>\gamma \}+\mu_{i}+\varepsilon_{i,t}\;i=1,...,n;t=1,...,T$$ (3)

where *q*_it_ is the threshold variable and *x*_it_ may include lagged dependent variables. It was predicated on the assumption that *T* remains constant as the sample size n approaches infinity. Consequently, we employ the generalized method moments (GMM) to estimate the unknown parameters *θ = β′, δ′,* and *γ′* and eliminate the incidental parameter *µ*_i_ using the first-difference transformation.

#### 3.3.2. Juodis, Karavias, and Sarafidis (2021) panel causality tests.

Juodis, Karavias [7] have recently developed a novel method for testing the null hypothesis of no Granger causality. This method is applicable to models with homogeneous or heterogeneous coefficients. The test is also known as the JKS panel non-causality test. It is possible to represent the test as in equation (4) as follows:


$$y_{i,t}=\lambda_{0,i}+\sum_{p=1}^{P}\lambda_{p,i}y_{i,t-p}+\sum_{p=1}^{P}\phi_{p,i}x_{i,t-p}+\varepsilon_{i,t}$$ (4)

where for *i* = 1;…; *N* and *t* = 1; …; *T*. In order to facilitate exposition and maintain generality, *x*_i,t_ is presumed that it is a scalar. The parameters $\lambda_{0,i}$ represent the individual-specific effects, $\varepsilon_{i,t}$ are the errors, $\lambda_{p,i}$ mean the heterogeneous autoregressive coefficients, while *p* = 1;...; *P*, and $\phi_{p,i}$ are the heterogeneous feedback coefficients, or Granger-causality parameters.

## 4. Empirical results

**Fig 1:**
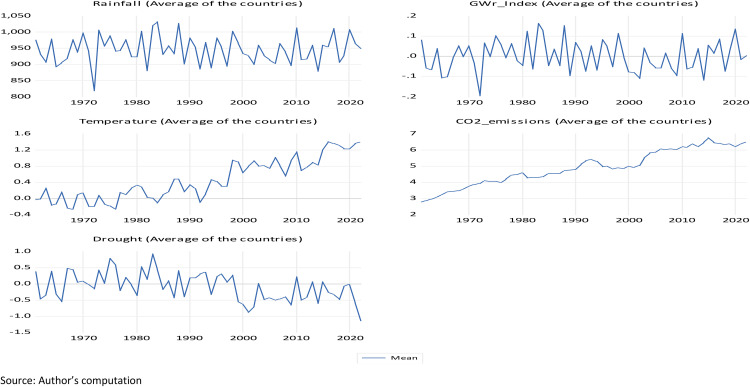
Graphical representations.

Fig 1 shows the graphical representations of the variables, such as Rainfall, GWr, Temperature, CO_2_ emissions, and Drought (SPEI index) for the panel of the countries over the period of 1961–2022. From the Fig, Rainfall and GWr generally move together, suggesting that higher precipitation corresponds to higher groundwater recharge. The SPEI index (drought measure) mirrors these patterns that rising during wetter periods and declining when rainfall and GWr fall. Conversely, temperature and CO_2_ emissions trend upward, shimmering global warming and industrial growth. Therefore, this Fig trend confirms that rainfall and groundwater recharge are crucial buffers against drought in the countries, while temperature rise and higher emissions reduce soil moisture and recharge which worsen drought conditions, whereas the parallel movement between rainfall, GWr, and SPEI supports the theoretical link between climate variability, water balance, and drought mitigation.

**Fig 2:**
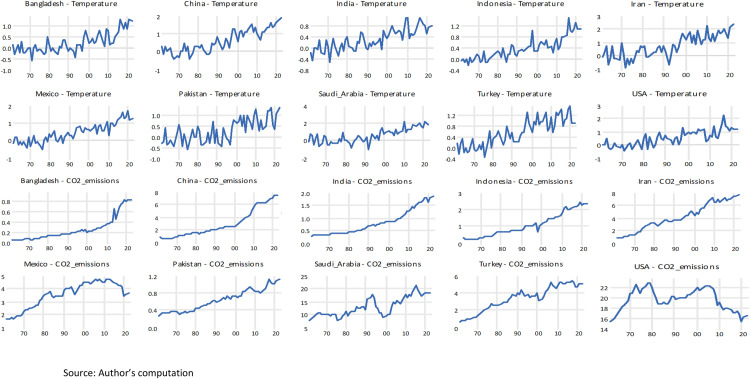
Graphical representations of Temperature and CO_2_ emissions for the Individual Countries.

As illustrated in Fig 2, the graphical representations of the variables are shown on average for the countries, including Temperature and CO_2_ emissions. These data are from the period between 1961 and 2022. Across all countries, temperature anomalies display a clear and persistent upward trajectory, indicating consistent warming regardless of regional, climatic, or economic differences. Countries such as China, India, Indonesia, Iran, Saudi Arabia, and Turkey exhibit particularly steep increases, while Bangladesh, Pakistan, Mexico, and the USA also show positive trends with greater year-to-year variability. The CO₂ emission patterns reveal substantial cross-country heterogeneity: China, India, Indonesia, and Iran demonstrate rapid and sustained emission growth; Pakistan and Bangladesh follow gradual upward paths; Mexico and Turkey stabilize or fluctuate after the mid-2000s; and the USA shows a notable decline in emissions after peaking around 2007. Despite these differences, all countries experience long-run temperature increases, suggesting that warming is influenced not only by national emission profiles but also by accumulated global GHG concentrations.

**Fig 3:**
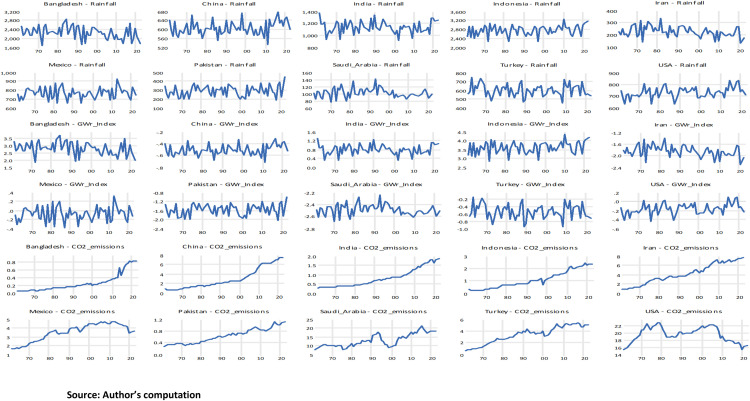
Graphical representations of Rainfall, GWr, and Drought for the Individual Countries.

In Fig 3, the graphical representations of the variables are shown on average for the countries, including Rainfall, GWr, and Drought (SPEI index). According to the Fig, rainfall levels across all countries exhibit substantial year-to-year variability but no strong or uniform long-term trend. Some countries, such as Indonesia, India, and Bangladesh, experience intermittent periods of higher rainfall, while others, including Iran, Turkey, and Mexico, show more irregular and fluctuating patterns. The GWr Index similarly display considerable variability but do not indicate a consistent directional trend; instead, most countries experience cyclical fluctuations, suggesting that groundwater availability is shaped by short-term hydrological conditions rather than sustained long-run improvements. In contrast, the CO₂ emissions series reveal distinct and persistent upward trajectories for most countries, particularly China, India, Indonesia, and Iran, reflecting rapid industrial growth and increasing fossil-fuel consumption. Countries such as Pakistan and Bangladesh show moderate but steady increases, whereas Mexico, Turkey, and especially the USA exhibit stabilization or declines in emissions in recent decades due to structural energy transitions.

As illustrated in Table 2, the descriptive statistics of the variables, including rainfall, GWr, temperature, CO_2_ emissions, and drought, are reported for the panel of countries. The data presented in the table indicates that the mean drought (SPEI) value of −0.091 signifies that, on average, the countries periodically experience mild drought conditions. Rainfall (mean 945 mm) exhibits significant variability (SD = 845 mm), indicative of climatic heterogeneity. The mean groundwater recharge (GWr) was found to be equal to zero, with a large dispersion (SD = 1.865), thereby indicating substantial variability across the countries. Temperature (mean deviation = 0.45°C) and CO₂ emissions (mean = 4.9 metric tons) exhibited moderately elevated levels, indicative of warming conditions. Therefore, the descriptive statistics reveal strong spatial heterogeneity, in which the countries differ widely in terms of hydrological capacity and exposure to drought. Furthermore, the high standard deviations in rainfall and GWr confirm the existence of nonlinear relationships and threshold effects between the water variables under study and drought.

As illustrated in Table 3, the study’s variables namely, rainfall, GWr, temperature, CO_2_ emissions, and drought for the individual countries are reported with their descriptive statistics. A study of the high rainfall and recharge countries (Indonesia and Bangladesh) reveals positive GWr values and higher mean SPEI, indicating a less severe drought. Conversely, arid countries such as Saudi Arabia, Iran, and Pakistan exhibit negative GWr indices, minimal rainfall, and amplified negative SPEI, indicative of more pronounced drought conditions. However, temperate countries (e.g., the USA, China, and Turkey) demonstrate moderate values. However, CO₂ emissions exhibit significant variation, ranging from 0.25 tons in Bangladesh to 19.6 tons in the USA. This suggests that hydrological endowments play a pivotal role in determining drought resilience, with countries exhibiting positive GWr and high rainfall demonstrating a capacity to maintain water security, while those with limited recharge experience recurrent drought. The contrast accentuates the pivotal function of groundwater in climate adaptation and sustainable drought management.

**Table 3:** Descriptive statistics for the individual countries.

	Obs.	Mean	Std. Dev.	Min	Max
**Bangladesh**					
Drought	62	−.37	1.02	−2.403	1.924
Rainfall	62	2241.549	274.728	1678.22	2843.91
Temperature	62	.233	.447	−.579	1.337
CO_2_ emissions	62	.251	.227	.046	.844
GWr index	62	2.807	.444	1.858	3.737
**China**					
Drought	62	−.022	1.072	−2.592	1.948
Rainfall	62	605.616	31.082	531.98	676.47
Temperature	62	.586	.601	−.408	1.906
CO_2_ emissions	62	2.869	2.141	.567	7.45
GWr index	62	−.51	.089	−.725	−.312
**India**					
Drought	62	.041	1.211	−2.284	2.717
Rainfall	62	1137.253	102.155	912.2	1373.3
Temperature	62	.299	.389	−.519	1.134
CO_2_ emissions	62	.797	.475	.284	1.87
GWr index	62	.789	.221	.285	1.28
**Indonesia**					
Drought	62	−.109	1.018	−2.312	1.755
Rainfall	62	2758.7	226.965	2237.01	3275.81
Temperature	62	.372	.404	−.257	1.488
CO_2_ emissions	62	1.059	.686	.227	2.46
GWr index	62	3.607	.336	2.811	4.346
**Iran**					
Drought	62	−.358	1.363	−5.875	2.548
Rainfall	62	224.13	42.849	128.95	335.79
Temperature	62	.698	.823	−.959	2.37
CO_2_ emissions	62	4.098	2.128	.741	7.67
GWr index	62	−1.845	.195	−2.323	−1.38
**Mexico**					
Rainfall	62	.074	1.026	−1.894	2.805
Temperature	62	763.942	61.639	651.2	929.81
CO_2_ emissions	62	.437	.529	−.495	1.726
GWr index	62	3.5	.99	1.59	4.73
**Pakistan**					
Drought	62	−.012	1.255	−5.138	1.691
Rainfall	62	288.689	60.192	181.5	442.88
Temperature	62	.291	.544	−.584	1.389
CO_2_ emissions	62	.622	.247	.268	1.1
GWr index	62	−1.574	.243	−2.038	−1.005
**Saudi Arabia**					
Drought	62	−.424	1.192	−3.261	2.103
Rainfall	62	102.202	13.211	74.56	141.3
Temperature	62	.528	.788	−.985	2.203
CO_2_ emissions	62	12.863	3.595	7.51	21.3
GWr index	62	−2.493	.087	−2.689	−2.252
**Turkey**					
Drought	62	−.052	1.144	−2.215	2.226
Rainfall	62	599.776	68.725	456.42	742.32
Temperature	62	.562	.496	−.35	1.547
CO_2_ emissions	62	3.302	1.436	.617	5.36
GWr index	62	−.53	.197	−.961	−.137
**USA**					
Drought	62	.316	.969	−1.952	2.275
Rainfall	62	731.165	45.343	631.09	837
Temperature	62	.49	.59	−.415	2.224
CO_2_ emissions	62	19.613	2.155	15.2	23.1
GWr index	62	−.168	.119	−.437	.103

Source: Author’s computation.

As illustrated in Table 4, an investigation into multicollinearity was conducted for Model 1, encompassing both the panel of countries and the individual countries, respectively. As illustrated in Panel A, which presents the correlation analysis of the panel of countries, there is a negative correlation between rainfall and temperature (−0.142). This finding suggests that, on average, periods of higher rainfall tend to coincide with slightly lower temperatures across the panel of countries. A study of the relationship between rainfall and CO_2_ emissions reveals a negative correlation (−0.355). This suggests that countries or periods with higher emissions experience slightly reduced rainfall. This finding is consistent with evidence linking emissions to changing precipitation patterns. A positive correlation has been identified between temperature and CO₂ emissions (0.234), suggesting a relationship in which elevated temperatures are associated with increased CO₂ emissions. This finding is consistent with the prevailing scientific consensus regarding the effects of greenhouse gases. However, with respect to multicollinearity, all correlation coefficients are below and within 0.70, suggesting no serious multicollinearity problem among the explanatory variables in the panel of model. This finding indicates that the inclusion of these variables within a single model does not result in instability of coefficient estimates. As illustrated in Panel B, which concerns the individual countries, it is evident that in most countries, there is a positive correlation (0.5–0.7) between CO₂ emissions and temperature. This finding is consistent with the principles of climate change theory. However, the relationship between rainfall and precipitation varies significantly across different regions. In tropical regions, such as China and Indonesia, there is a positive correlation between rainfall and precipitation. In contrast, in arid or semi-arid regions, including Iran and Saudi Arabia, there is a negative correlation between these two variables. Furthermore, the pair of independent variables are all below or within 0.70, which confirms the absence of multicollinearity.

**Table 4:** Correlation analysis for model 1.

*Panel A: For the panel of the countries*	(1)	(2)	(3)
(1) Rainfall	1.000		
(2) Temperature	−0.142	1.000	
(3) CO_2_ emissions	−0.355	0.234	1.000
*Panel B: For the individual countries*			
**Bangladesh**			
(1) Rainfall	1.000		
(2) Temperature	−0.233	1.000	
(3) CO_2_ emissions	−0.306	0.684	1.000
**China**			
(1) Rainfall	1.000		
(2) Temperature	0.264	1.000	
(3) CO_2_ emissions	0.271	0.541	1.000
**India**			
(1) Rainfall	1.000		
(2) Temperature	−0.179	1.000	
(3) CO_2_ emissions	0.017	0.530	1.000
**Indonesia**			
(1) Rainfall	1.000		
(2) Temperature	0.277	1.000	
(3) CO_2_ emissions	0.260	0.545	1.000
**Iran**			
(1) Rainfall	1.000		
(2) Temperature	−0.506	1.000	
(3) CO_2_ emissions	−0.287	0.576	1.000
**Mexico**			
(1) Rainfall	1.000		
(2) Temperature	−0.081	1.000	
(3) CO_2_ emissions	0.095	0.673	1.000
**Pakistan**			
(1) Rainfall	1.000		
(2) Temperature	−0.101	1.000	
(3) CO_2_ emissions	0.139	0.616	1.000
**Saudi Arabia**			
(1) Rainfall	1.000		
(2) Temperature	−0.364	1.000	
(3) CO_2_ emissions	−0.105	0.634	1.000
**Turkey**			
(1) Rainfall	1.000		
(2) Temperature	0.023	1.000	
(3) CO_2_ emissions	−0.087	0.681	1.000
**USA**			
(1) Rainfall	1.000		
(2) Temperature	0.167	1.000	
(3) CO_2_ emissions	−0.083	−0.162	1.000

Source: Author’s computation

The climate-emission linkage is robust, especially for temperature. Regional variations highlight how local geography mediates rainfall responses. Empirical models using these variables can proceed safely without fear of multicollinearity bias. Furthermore, CO₂ mitigation could help moderate temperature rises and stabilize rainfall in emission-sensitive economies.

As illustrated in Table 5, the correlation analysis results are reported. These results indicate the presence or absence of multicollinearity among the explanatory variables. These explanatory variables were utilized in Model 2. The analysis was conducted for both the panel and individual countries, respectively. The data presented in the table indicates a negative correlation between the groundwater recharge (GWr) and the temperature of −0.151. This finding suggests that an increase in temperature is associated with a decrease in groundwater recharge. Furthermore, the observed negative GWr–CO₂ correlation (−0.363) suggests a linkage between elevated emissions and diminished groundwater recharge, indicating that emissions may amplify hydrological stress. Furthermore, the Temperature- CO_2_ (0.234) index persists in a positive state, thereby validating the emission-driven warming pattern. However, with respect to multicollinearity, all correlations are below or within 0.70, indicating that multicollinearity is not a problem among the explanatory variables in Model 2. As illustrated in Panel B, which analyzes the relationship between CO_2_ and temperature at the country level in Model 2, a high correlation (0.60–0.70) is observed across countries. This finding is consistent with the results observed in Model 1. Generally, the growth rate (GWr) of a system tends to decrease as the temperature and emissions levels of that system increase. However, there are a few exceptions to this rule. These exceptions are tropical countries, such as China and Indonesia. However, an examination of multicollinearity among the explanatory variables for each country revealed no such issues, thereby validating the model specification and confirming the retention of the explanatory variables within the same model.

**Table 5:** Correlation analysis for model 2.

*Panel A: For the panel of the countries*	(1)	(2)	(3)
(1) GWr	1.000		
(2) Temperature	−0.151	1.000	
(3) CO_2_ emissions	−0.363	0.234	1.000
*Panel B: For the individual countries*			
**Bangladesh**			
(1) GWr	1.000		
(2) Temperature	−0.234	1.000	
(3) CO_2_ emissions	−0.311	0.704	1.000
**China**			
(1) GWr	1.000		
(2) Temperature	0.261	1.000	
(3) CO_2_ emissions	0.268	0.703	1.000
**India**			
(1) GWr	1.000		
(2) Temperature	−0.178	1.000	
(3) CO_2_ emissions	0.018	0.670	1.000
**Indonesia**			
(1) GWr	1.000		
(2) Temperature	0.276	1.000	
(3) CO_2_ emissions	0.258	0.605	1.000
**Iran**			
(1) GWr	1.000		
(2) Temperature	−0.499	1.000	
(3) CO_2_ emissions	−0.287	0.616	1.000
**Mexico**			
(1) GWr	1.000		
(2) Temperature	−0.080	1.000	
(3) CO_2_ emissions	0.094	0.673	1.000
**Pakistan**			
(1) GWr	1.000		
(2) Temperature	−0.115	1.000	
(3) CO_2_ emissions	0.129	0.626	1.000
**Saudi Arabia**			
(1) GWr	1.000		
(2) Tem**pe**rature	−0.353	1.000	
(3) CO_2_ emissions	−0.100	0.634	1.000
**Turkey**			
(1) GWr	1.000		
(2) Temperature	0.027	1.000	
(3) CO_2_ emissions	−0.086	0.681	1.000
**USA**			
(1) GWr	1.000		
(2) Temperature	0.165	1.000	
(3) CO_2_ emissions	−0.080	−0.162	1.000

Source: Author’s computation

The increasing levels of emissions and temperatures are contributing to a reduction in groundwater recharge, particularly in arid regions such as Iran, Saudi Arabia, and Bangladesh. In contrast, countries with tropical climates, including China and Indonesia, may experience short-term benefits from heightened rainfall intensity. However, the long-term effects in these regions are likely to be negative. Therefore, it is imperative to integrate emission control measures with water management strategies. Furthermore, the absence of multicollinearity in the regression model ensures the reliability of coefficient estimates, which link groundwater recharge to emissions and temperature.

Table 6 presents the results of the Dynamic Panel Threshold Regression (DPThR) model regarding the relationship between rainfall and drought. According to the table, rainfall is positively related with the index of SPEI (drought is represented by SPEI index in which increase in this index suggests reducing drought risk, i.e., the higher the value of the index, the wetter the conditions) at 1% significance level, indicating that a 1-millimeter increase in rainfall increase the SPEI index by 0.003. In other words, increase in rainfall is effectively lowering the likelihood of drought in overexploited groundwater regions. Temperature is negatively related with the index of SPEI and the impact is significant at 1% level, i.e., increase in temperature is really raising drought where a 1°C increase in temperature induces a decline in SPEI by 0.68 SPEI. Thus, increased temperature is significantly raising the likelihood of drought in overexploited groundwater regions. CO_2_ emissions is also negatively related with the index of SPEI, i.e., increase in CO_2_ emissions is raising drought where a 1-metric ton increase in CO_2_ emissions per capita declines the SPEI index by 0.03, indicating that higher carbon emissions increase the likelihood of drought though the impact is insignificant. However, the threshold level of rainfall required to mitigate the menace of drought is 614.41 millimeters. The rainfall threshold represents a critical point beyond which the mitigation of droughts becomes more pronounced. Specifically, rainfall has a threshold-dependent effect: when precipitation falls below 614 mm, the risk of drought increases significantly, whereas above this threshold, moisture replenishment enhances drought resilience. Nevertheless, temperature remains the primary climatic stressor in these countries.

**Table 6:** Result of DPThR on the panel countries for model 1.

Drought	Coefficient	Std. Err.	z	P > z
Rainfall	.0027693	.0004141	6.69	0.000*
Temperature	−.6791567	.0718164	−9.46	0.000*
CO_2_ emissions	−.0276834	.0237671	−1.16	0.244
Cons	−3.62973	.2986528	−12.15	0.000*
Threshold	614.41			

Source: Author’s computation

Note: * indicates significance at 1% level.

The results of the DPThR model regarding the relationship between groundwater recharge (GWr) and drought are presented in Table 7. Following the table, GWr is positively related with the index of SPEI and it is significant at 1%, i.e., increase in GWr is effectively lowering drought where a 1-standard deviation of groundwater recharge will reduce drought by 5.06 SPEI. In other words, an increase in groundwater recharge decreases the likelihood of drought. Temperature is negatively related with the index of SPEI and it is significant at 1%, i.e., increase in temperature led to raising drought where a 1°C increase in temperature will cause a rise in drought index by 0.49 SPEI. Thus, the rise in temperature is influencing the occurrence of drought, which is consistent with the findings in Table 7 regarding temperature. CO_2_ emissions is also negatively related with the index of SPEI, i.e., increase in CO_2_ emissions is raising drought where a 1-metric ton increase in CO_2_ emissions per capita is raising drought index by 0.03 SPEI, though the impact is insignificant. This finding reinforces the result from Table 7 regarding CO_2_ emissions.

**Table 7:** Result of DPThR on the panel countries for model 2.

Drought	Coefficient	Std. Err.	z	P > z
GW_r_	5.06448	.2062461	24.56	0.000*
Temperature	−.4883108	.0567497	−8.60	0.000*
CO_2_ emissions	−.0287845	.0195335	−1.47	0.141
Cons	2.536354	.1997627	12.70	0.000*
Threshold	−.0039			

Source: Author’s computation

Note that, * indicates significance at 1% level

The threshold level of groundwater recharge (GWr) required to mitigate the effects of drought is identified as −0.0039 standard deviation. This threshold signifies the point beyond which the alleviation of drought intensifies. Groundwater recharge exhibits a threshold-dependent effect: when recharge falls below −0.0039 standard deviation, the risk of drought increases rapidly; conversely, when recharge exceeds this threshold, moisture replenishment enhances drought resilience. The findings underscore the critical role of groundwater recharge as a powerful mitigating factor against drought, where even small improvements in recharge levels can significantly reduce drought severity. However, the low threshold value indicates a high sensitivity of drought response to variations in groundwater availability.

**Fig 4:**
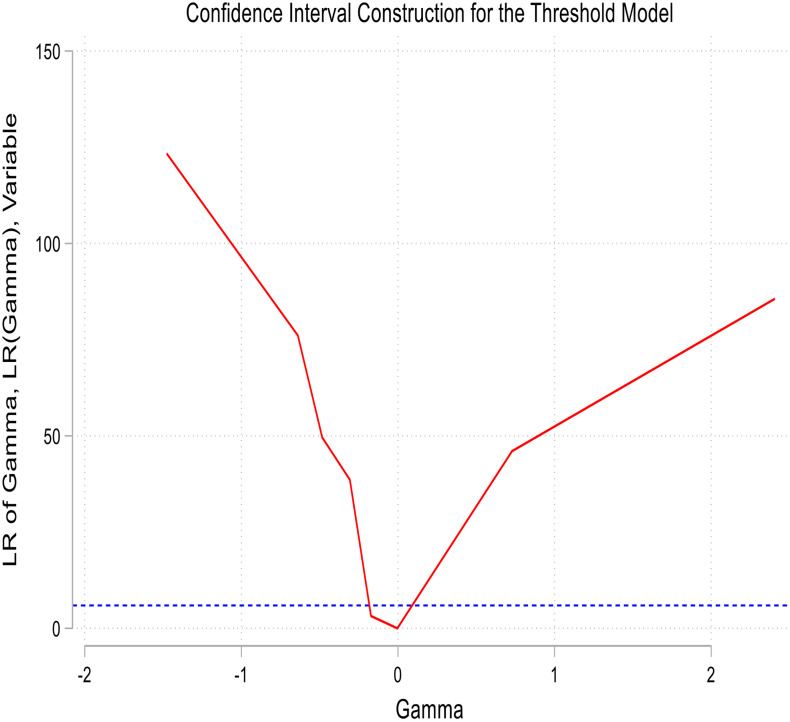
Threshold graph on the panel for model 1.

**Fig 5:**
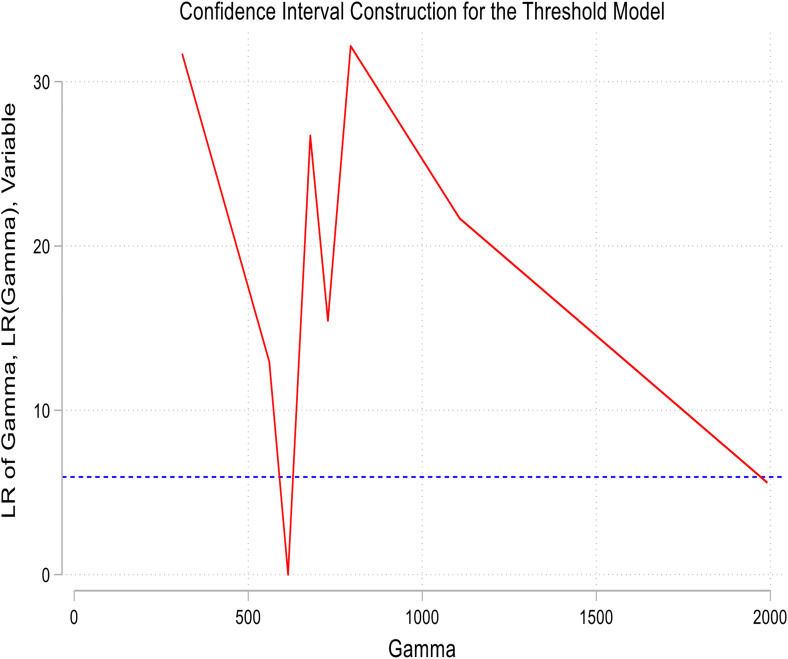
Threshold graph on the panel for model 2.

Figs 4 and 5 present the threshold graphs for the panel of countries in relation to Models 1 and 2. Both graphs exhibit distinct nonlinear patterns. Specifically, when rainfall is below 614 mm or groundwater recharge (GWr) is below −0.0039 standard deviation, the Standardized Precipitation Evapotranspiration Index (SPEI) declines sharply, indicating a worsening of drought conditions. Beyond these thresholds, the slope of the graph’s levels off, reflecting stabilization and a reduction in drought risk. These observations confirm two critical environmental control points: first, the adequacy of rainfall and sufficiency of groundwater recharge; and second, the importance of maintaining water balance beyond these levels to mitigate the risk of recurrent droughts in climate-sensitive economies.

Table 8 presents the results of the Drought Threshold Response (DThR) for Model 1 across individual countries. The table demonstrates that rainfall exerts a positive and significant effect on the Standardized Precipitation Evapotranspiration Index (SPEI) for most countries (Bangladesh, China, India, Iran, Mexico, Saudi Arabia, Turkey, and the USA) at various conventional levels of significance, indicating that higher rainfall mitigates drought conditions. In contrast, temperature consistently exhibits a negative effect on drought, with significant impacts across nearly all cases, suggesting that rising temperatures exacerbate drought. Regarding CO_2_ emissions, the effect is generally insignificant, except in Bangladesh, where a positive relationship is observed, pointing to localized emission–moisture interactions. The rainfall thresholds vary considerably, ranging from 99 mm in Saudi Arabia (arid) to 2877 mm in Indonesia (humid). This variability underscores that each country has a unique rainfall–drought threshold, which is shaped by its climatic zone. Arid regions, where even modest rainfall can aid drought recovery, contrast with humid regions that require more substantial rainfall deficits before droughts emerge. These findings emphasize the spatial heterogeneity of drought vulnerability.

**Table 8:** Result of DThR on the individual countries for model 1.

	Coefficient	Std. Err.	z	P > z
**Bangladesh**				
Rainfall	.0032602	.0000801	40.71	0.000*
Temperature	−.191303	.0473092	−4.04	0.000*
CO_2_ emissions	.2504787	.0965703	2.59	0.009*
Threshold	2367.37			
**China**				
Rainfall	.0397684	.0029227	13.61	0.000*
Temperature	−.7215681	.1577455	−4.57	0.000*
CO_2_ emissions	.0053081	.0463244	0.11	0.909
Threshold	612.52			
**India**				
Rainfall	.0071907	.001676	4.29	0.000*
Temperature	.4715368	.567058	0.83	0.406
CO_2_ emissions	.3139121	.4575173	0.69	0.493
Threshold	1266.10			
**Indonesia**				
Rainfall	.0001858	.0007867	0.24	0.813
Temperature	.5525684	.5716264	0.97	0.334
CO_2_ emissions	−.2830246	.3347702	−0.85	0.398
Threshold	2876.96			
**Iran**				
Rainfall	.0193692	.0022206	8.72	0.000*
Temperature	−.7042626	.1528215	−4.61	0.000*
CO_2_ emissions	.0452805	.0534619	0.85	0.397
Threshold	176.58			
**Mexico**				
Rainfall	.011455	.0014758	7.76	0.000*
Temperature	−.3277618	.1591707	−2.06	0.039**
CO_2_ emissions	−.0531409	.085369	−0.62	0.534
Threshold	723.59			
**Pakistan**				
Rainfall	.0032415	.0033008	0.98	0.326
Temperature	−1.229351	.2962837	−4.15	0.000*
CO_2_ emissions	.7739861	.6613651	1.17	0.242
Threshold	264.72			
**Saudi Arabia**				
Rainfall	.0310021	.0064535	4.80	0.000*
Temperature	−1.12625	.0909582	−12.38	0.000*
CO_2_ emissions	.0349054	.0194097	1.80	0.072***
Threshold	99.46			
**Turkey**				
Rainfall	.0130207	.0012218	10.66	0.000*
Temperature	−.4345841	.1574532	−2.76	0.006*
CO_2_ emissions	−.005915	.0552514	−0.11	0.915
Threshold	622.94			
**USA**				
Rainfall	.0188075	.0014287	13.16	0.000*
Temperature	−.4062646	.0725499	−5.60	0.000*
CO_2_ emissions	.0173321	.0198265	0.87	0.382
Threshold	718.26			

Source: Author’s computation

Note that, *, **, and *** indicate significance at 1%, 5%, and 10% levels, respectively.

Table 9 presents the results of the Drought Threshold Response (DThR) for Model 2 across individual countries. The table shows that the groundwater recharge (GWr) coefficient is positive and significant in nearly all countries at conventional levels of significance, with the exception of Pakistan, suggesting that increased groundwater recharge helps mitigate drought conditions. Temperature remains negatively correlated with groundwater recharge and is highly significant, indicating that warming reduces recharge and exacerbates drought. CO₂ emissions generally exhibit an insignificant relationship with drought at conventional significance levels, except in Bangladesh, where a positive association with improved SPEI is observed, possibly due to industrial irrigation or recharge initiatives. Groundwater recharge thresholds vary, with low thresholds (−2.54 in Saudi Arabia) in arid regions and higher thresholds (+3.23 in Indonesia) in humid regions. These thresholds define each country’s hydrological resilience limit, with arid countries experiencing rapid drought onset when recharge levels decrease slightly, while humid regions can sustain drought conditions for longer periods. Consequently, effective groundwater management emerges as a key strategic lever for drought mitigation.

**Table 9:** Result of DThR on the individual countries for model 2.

	Coefficient	Std. Err.	z	P > z
**Bangladesh**				
GWr	1.955113	.0544747	35.89	0.000*
Temperature	−.2071154	.0534224	−3.88	0.000*
CO_2_ emissions	.2712045	.1091998	2.48	0.013**
Threshold	3.02			
**China**				
GWr	13.87326	1.019831	13.60	0.000*
Temperature	−.7192549	.1577562	−4.56	0.000*
CO_2_ emissions	.0053217	.0463333	0.11	0.909
Threshold	−0.49			
**India**				
GWr	3.286448	.7659096	4.29	0.000*
Temperature	.4721008	.5670672	0.83	0.405
CO_2_ emissions	.3103615	.4576478	0.68	0.498
Threshold	1.06			
**Indonesia**				
GWr	2.004366	.5174046	3.87	0.000*
Temperature	.558542	.5716228	0.98	0.329
CO_2_ emissions	−.3316817	.3367153	−0.99	0.325
Threshold	3.23			
**Iran**				
GWr	4.169074	.5866919	7.11	0.000*
Temperature	−.6931083	.1528541	−4.53	0.000*
CO_2_ emissions	.0350793	.0534673	0.66	0.512
Threshold	−1.78			
**Mexico**				
GWr	4.526305	.5845587	7.74	0.000*
Temperature	−.3325807	.1592839	−2.09	0.037**
CO_2_ emissions	−.0511101	.0854292	−0.60	0.550
Threshold	−0.19			
**Pakistan**				
GWr	1.081997	.8403037	1.29	0.198
Temperature	−1.198167	.2963606	−4.04	0.000*
CO_2_ emissions	.7044392	.6592986	1.07	0.285
Threshold	−1.66			
**Saudi Arabia**				
GWr	6.903984	.8878542	7.78	0.000*
Temperature	−1.132997	.0902522	−12.55	0.000*
CO_2_ emissions	.030009	.0186603	1.61	0.108
Threshold	−2.54			
**Turkey**				
GWr	4.489684	.4194203	10.70	0.000*
Temperature	−.447794	.1571761	−2.85	0.004*
CO_2_ emissions	−.0035992	.0551345	−0.07	0.948
Threshold	−0.46			
**USA**				
GWr	8.267093	.5034463	16.42	0.000*
Temperature	−.4341873	.0744464	−5.83	0.000*
CO_2_ emissions	.0098725	.0197032	0.50	0.616
Threshold	−0.26			

Source: Author’s computation

Note that, *, **, and *** indicate significance at 1%, 5%, and 10% levels, respectively.

Table 10 presents the results of the Juodis, Karavias (7) panel non-causality test across the countries for Models 1 and 2. The table indicates that rainfall, groundwater recharge (GWr), CO_2_ emissions, and temperature all Granger-cause drought at conventional levels of significance. However, drought does not significantly Granger-cause these variables, except for weak feedback to CO₂ emissions. This causal direction confirms that climatic and hydrological changes drive drought, rather than drought acting as a precursor to these variables. Thus, drought is an outcome of climatic imbalance, not a cause. The results from the JKS panel non-causality test provide a robustness check for the findings obtained from the DPThR model. The consistency of the results from both the methods reinforces the robustness of the study’s findings.

**Table 10:** Result of the JKS Panel Non-causality Test on the panel countries for models 1 and 2.

Null Hypothesis:	HPJ Wald test	Prob.
*Rainfall* does Granger-cause *Drought* for at least one panelvar	24.934	0.0213**
*Drought* does Granger-cause *Rainfall* for at least one panelvar	2.2626	0.1329
*GW*_*r*_ does Granger-cause *Drought* for at least one panelvar	15.865	0.0000*
*Drought* does Granger-cause *GW*_*r*_ for at least one panelvar	1.3540	0.2446
*CO*_*2*_ *emissions* does Granger-cause *Drought* for at least one panelvar	25.113	0.0000*
*Drought* does Granger-cause *CO*_*2*_ *emissions* for at least one panelvar	24.315	0.0903***
*Temperature* does Granger-cause *Drought* for at least one panelvar	12.227	0.0005*
*Drought* does Granger-cause *Temperature* for at least one panelvar	2.2771	0.1313

*, **, and *** symbolize level of significance at 1%, 5%, and 10%, respectively.

Table 11 presents the results of the Juodis, Karavias (7) non-causality test for individual countries under Models 1 and 2. The table shows that in nearly all countries, rainfall and groundwater recharge (GWr) Granger-cause drought, confirming a strong hydro-climatic control. Several countries exhibit bidirectional causality, suggesting feedback loops, where drought may reduce soil infiltration, subsequently lowering future GWr and the efficiency of rainfall. CO₂ emissions are found to significantly Granger-cause drought in industrializing countries, such as China, India, and Iran, but not in more stable economies like the USA and Mexico. Temperature consistently Granger-causes drought across all countries. These findings highlight the existence of hydro-climatic feedback loops: low recharge and rainfall contribute to drought, and drought further weakens the hydrological cycle. This cyclical relationship underscores the need for integrated climate and water resource management policies that are tailored to each nation’s specific water threshold dynamics. Furthermore, the results from the JKS non-causality test serve as a robustness check for the conclusions derived from the DThR model. The consistency of the findings across both methods reinforces the reliability and robustness of the study’s results.

**Table 11:** Result of the JKS Non-causality Test on the individual countries for models 1 and 2.

	HPJ Wald test	Prob.
**Bangladesh**		
*Rainfall* does Granger-cause *Drought* for at least one panelvar	2.2e + 25	0.0000*
*Drought* does Granger-cause *Rainfall* for at least one panelvar	2.7e + 24	0.0000*
*GW*_*r*_ does Granger-cause *Drought* for at least one panelvar	1.3e + 25	0.0000*
*Drought* does Granger-cause *GW*_*r*_ for at least one panelvar	2.0e + 24	0.0000*
*CO*_*2*_ *emissions* does Granger-cause *Drought* for at least one panelvar	1.9e + 32	0.0000*
*Drought* does Granger-cause *CO*_*2*_ *emissions* for at least one panelvar	1.8e + 28	0.0000*
*Temperature* does Granger-cause *Drought* for at least one panelvar	12.6397	0.0000*
*Drought* does Granger-cause *Temperature* for at least one panelvar	7.9e + 30	0.0000*
**China**		
*Rainfall* does Granger-cause *Drought* for at least one panelvar	2.1e + 27	0.0000*
*Drought* does Granger-cause *Rainfall* for at least one panelvar	2.6e + 26	0.0000*
*GW*_*r*_ does Granger-cause *Drought* for at least one panelvar	7.2e + 29	0.0000*
*Drought* does Granger-cause *GW*_*r*_ for at least one panelvar	2.2e + 29	0.0000*
*CO*_*2*_ *emissions* does Granger-cause *Drought* for at least one panelvar	9.1250	0.0025*
*Drought* does Granger-cause *CO*_*2*_ *emissions* for at least one panelvar	5.6e + 29	0.0000*
*Temperature* does Granger-cause *Drought* for at least one panelvar	7.7e + 31	0.0000*
*Drought* does Granger-cause *Temperature* for at least one panelvar	11.7659	0.0006*
**India**		
*Rainfall* does Granger-cause *Drought* for at least one panelvar	3.9e + 27	0.0000*
*Drought* does Granger-cause *Rainfall* for at least one panelvar	5.3e + 29	0.0000*
*GW*_*r*_ does Granger-cause *Drought* for at least one panelvar	3.0e + 28	0.0000*
*Drought* does Granger-cause *GW*_*r*_ for at least one panelvar	5.8e + 29	0.0000*
*CO*_*2*_ *emissions* does Granger-cause *Drought* for at least one panelvar	13.8059	0.0002*
*Drought* does Granger-cause *CO*_*2*_ *emissions* for at least one panelvar	1.9e + 31	0.0000*
*Temperature* does Granger-cause *Drought* for at least one panelvar	5.7e + 31	0.0000*
*Drought* does Granger-cause *Temperature* for at least one panelvar	1.3e + 27	0.0006*
**Indonesia**		
*Rainfall* does Granger-cause *Drought* for at least one panelvar	2.6e + 29	0.0000*
*Drought* does Granger-cause *Rainfall* for at least one panelvar	3.4e + 26	0.0000*
*GW*_*r*_ does Granger-cause *Drought* for at least one panelvar	1.4e + 30	0.0000*
*Drought* does Granger-cause *GW*_*r*_ for at least one panelvar	7.9e + 24	0.0000*
*CO*_*2*_ *emissions* does Granger-cause *Drought* for at least one panelvar	7.3e + 29	0.0000*
*Drought* does Granger-cause *CO*_*2*_ *emissions* for at least one panelvar	0.0925	0.7610
*Temperature* does Granger-cause *Drought* for at least one panelvar	3.9e + 29	0.0000*
*Drought* does Granger-cause *Temperature* for at least one panelvar	7.2e + 31	0.0000*
**Iran**		
*Rainfall* does Granger-cause *Drought* for at least one panelvar	5.2e + 28	0.0000*
*Drought* does Granger-cause *Rainfall* for at least one panelvar	5.0e + 26	0.0000*
*GW*_*r*_ does Granger-cause *Drought* for at least one panelvar	3.8e + 28	0.0000*
*Drought* does Granger-cause *GW*_*r*_ for at least one panelvar	5.9e + 26	0.0000*
*CO*_*2*_ *emissions* does Granger-cause *Drought* for at least one panelvar	2.8e + 33	0.0000*
*Drought* does Granger-cause *CO*_*2*_ *emissions* for at least one panelvar	29.3695	0.7610
*Temperature* does Granger-cause *Drought* for at least one panelvar	1.0e + 33	0.0000*
*Drought* does Granger-cause *Temperature* for at least one panelvar	4.4e + 30	0.0000*
**Mexico**		
*Rainfall* does Granger-cause *Drought* for at least one panelvar	3.6e + 28	0.0000*
*Drought* does Granger-cause *Rainfall* for at least one panelvar	5.6e + 24	0.0000*
*GW*_*r*_ does Granger-cause *Drought* for at least one panelvar	3.3e + 30	0.0000*
*Drought* does Granger-cause *GW*_*r*_ for at least one panelvar	0.0511	0.8112
*CO*_*2*_ *emissions* does Granger-cause *Drought* for at least one panelvar	6.7e + 26	0.0000*
*Drought* does Granger-cause *CO*_*2*_ *emissions* for at least one panelvar	0.4509	0.5019
*Temperature* does Granger-cause *Drought* for at least one panelvar	1.2e + 33	0.0000*
*Drought* does Granger-cause *Temperature* for at least one panelvar	5.6e + 31	0.0000*
**Pakistan**		
*Rainfall* does Granger-cause *Drought* for at least one panelvar	7.8e + 27	0.0000*
*Drought* does Granger-cause *Rainfall* for at least one panelvar	1.2e + 26	0.0000*
*GW*_*r*_ does Granger-cause *Drought* for at least one panelvar	6.0e + 28	0.0000*
*Drought* does Granger-cause *GW*_*r*_ for at least one panelvar	1.6e + 26	0.0000*
*CO*_*2*_ *emissions* does Granger-cause *Drought* for at least one panelvar	3.2682	0.0706***
*Drought* does Granger-cause *CO*_*2*_ *emissions* for at least one panelvar	1.7e + 29	0.0000*
*Temperature* does Granger-cause *Drought* for at least one panelvar	1.1e + 32	0.0000*
*Drought* does Granger-cause *Temperature* for at least one panelvar	1.8e + 31	0.0000*
**Saudi Arabia**		
*Rainfall* does Granger-cause *Drought* for at least one panelvar	13.9783	0.0002*
*Drought* does Granger-cause *Rainfall* for at least one panelvar	3.1e + 26	0.0000*
*GW*_*r*_ does Granger-cause *Drought* for at least one panelvar	3.2e + 27	0.0000*
*Drought* does Granger-cause *GW*_*r*_ for at least one panelvar	3.9e + 23	0.0000*
*CO*_*2*_ *emissions* does Granger-cause *Drought* for at least one panelvar	2.6e + 31	0.0000*
*Drought* does Granger-cause *CO*_*2*_ *emissions* for at least one panelvar	1.3e + 28	0.0000*
*Temperature* does Granger-cause *Drought* for at least one panelvar	1.9e + 31	0.0000*
*Drought* does Granger-cause *Temperature* for at least one panelvar	2.4e + 29	0.0000*
**Turkey**		
*Rainfall* does Granger-cause *Drought* for at least one panelvar	1.2e + 29	0.0000*
*Drought* does Granger-cause *Rainfall* for at least one panelvar	7.3e + 25	0.0000*
*GW*_*r*_ does Granger-cause *Drought* for at least one panelvar	3.0091	0.0828***
*Drought* does Granger-cause *GW*_*r*_ for at least one panelvar	9.4e + 26	0.0000*
*CO*_*2*_ *emissions* does Granger-cause *Drought* for at least one panelvar	5.0e + 31	0.0000*
*Drought* does Granger-cause *CO*_*2*_ *emissions* for at least one panelvar	3.1e + 28	0.0000*
*Temperature* does Granger-cause *Drought* for at least one panelvar	1.3e + 31	0.0000*
*Drought* does Granger-cause *Temperature* for at least one panelvar	2.0e + 27	0.0000*
**USA**		
*Rainfall* does Granger-cause *Drought* for at least one panelvar	6.5e + 26	0.0000*
*Drought* does Granger-cause *Rainfall* for at least one panelvar	0.6994	0.4030
*GW*_*r*_ does Granger-cause *Drought* for at least one panelvar	1.0e + 29	0.0000*
*Drought* does Granger-cause *GW*_*r*_ for at least one panelvar	1.5e + 30	0.0000*
*CO*_*2*_ *emissions* does Granger-cause *Drought* for at least one panelvar	3.0e + 27	0.0000*
*Drought* does Granger-cause *CO*_*2*_ *emissions* for at least one panelvar	1.1e + 27	0.0000*
*Temperature* does Granger-cause *Drought* for at least one panelvar	3.1e + 33	0.0000*
*Drought* does Granger-cause *Temperature* for at least one panelvar	3.9e + 31	0.0000*

*, **, and *** symbolize level of significance at 1%, 5%, and 10%, respectively.

## 5. Discussion

The results presented that an increase in rainfall reduces the likelihood of drought in the countries. This finding is consistent with Wilhite, Sivakumar [49], who emphasize the close connection between rainfall and drought, with rainfall playing a crucial role in either mitigating or exacerbating drought conditions. It also aligns with Trenberth, Dai [10], who argue that rainfall variability is a primary driver of droughts, and Dai, Zhao [50], who found that changes in rainfall patterns can influence drought frequency and severity. Liu, Gudmundsson [51] further support this, suggesting that rainfall intensity and duration can impact drought recovery. Furthermore, the threshold level of rainfall capable of mitigating drought effects is above 228.45 millimeters, which aligns with the Masson-Delmotte, Zhai [9], which states that droughts occur when rainfall is below average, leading to water scarcity and impacts on ecosystems and human societies. Therefore, among the top 10 countries with underground water overexploitation, the countries that are most at risk of experiencing drought due to low rainfall are Pakistan, Iran, and Saudi Arabia. These countries should be aware that whenever the level of rainfall falls below 614.41 millimeters, drought is likely to occur. This study provides novel insights by identifying specific thresholds for these countries, offering a more tailored approach compared to broader studies on rainfall-drought relationships. However, the causality test results in relation to rainfall confirm the findings of Trenberth, Dai [10], Dai, Zhao [50], and Liu, Gudmundsson [51] that increased rainfall reduces the occurrence of drought. This contrasts with the views of Seneviratne, Corti [15], Hirschi, Seneviratne [52], and Berg, Lintner [53], who argue that drought conditions also influence rainfall patterns. The discrepancy in findings may arise due to the regional focus of this study, which specifically examines countries with significant groundwater overexploitation, where rainfall has a more direct and observable impact on drought severity. This is in contrast to global-scale studies where the relationship between rainfall and drought is often averaged out, masking localized patterns and buffering effects. Likewise, the findings are consistent with those of Adib, Moradi [54], who established an interlinked relationship between meteorological, hydrological, and groundwater resource droughts, influenced by upstream reservoir regulation. This aligns with the current study’s observation that climatic and hydrological changes, including groundwater recharge, are integral drivers of drought, further reinforcing the interconnectedness of these factors in shaping drought outcomes.

Furthermore, the results reported that an increase in groundwater recharge reduces the likelihood of drought in the countries. This finding is consistent with McEvoy, Famiglietti [29], who suggest that higher groundwater recharge can mitigate drought effects. Henao Casas, Fernández Escalante [55] further support this, demonstrating that groundwater recharge alleviates drought impacts. However, when considering the panel as there is no evidence of causality running from drought to groundwater recharge but the other way round, this finding contradicts the views of Guo, Dirmeyer [30], who found that drought conditions can lead to a decrease in groundwater recharge. Moreover, Zaerpour, Hatami [56] demonstrated that climate-driven variations in baseflow significantly influence groundwater levels and drought intensity, emphasizing that shifts in temperature and precipitation patterns can alter subsurface flow contributions and, consequently, drought resilience. But in the case of the individual countries’ causality, the drought is causing groundwater recharge. This study finding on groundwater recharge lowering drought, suggests that enhancing groundwater recharge can help mitigate the effects of drought, regardless of the occurrence of drought conditions. However, Jasechko, Seybold [57] cautioned that in many regions, rivers and streams lose substantial amounts of water to underlying aquifers, which can diminish surface water availability and complicate groundwater recharge management, particularly during prolonged dry periods. The threshold level of groundwater recharge needed to mitigate drought is above −0.0039 standard deviation. Therefore, countries at risk of drought due to insufficient groundwater recharge among the top 10 countries with underground water overexploitation include China, Iran, Mexico, Pakistan, Saudi Arabia, Turkey, and USA. These countries should be mindful that when the level of groundwater recharge falls below −0.0039 standard deviation, they are at risk of drought. Furthermore, the causality test results support a one-way relationship, confirming that groundwater recharge is a key determinant in mitigating drought, rather than being influenced by drought conditions. This is a crucial finding, as it highlights the importance of proactive water management strategies to ensure sufficient groundwater recharge in drought-prone areas.

Moreover, the results indicate that an increase in CO_2_ emissions raises the likelihood of drought in the countries but the effect is weak considering that its increase is positive with drought (lowering SPEI). Nonetheless, the positivity finding is consistent with Trenberth, Dai [10], who argue that increased CO_2_ emissions alter precipitation patterns, as well as with Seneviratne, Corti [15], Reichstein, Bahn [58], and Schwalm, Williams [59], who note that higher CO_2_ emissions increase evaporation, water loss, and temperature extremes, all of which contribute to drought. Bowman, Williamson [60] and Houghton, House [61] also suggest that carbon emissions influence drought. The threshold level of CO_2_ emissions required to mitigate the effects of drought is below 19.34 metric tons per capita. This suggests that the USA and Mexico, among the top 10 countries with ground water overexploitation, are particularly at risk of experiencing drought when CO_2_ emissions exceed this threshold. The causality test results in relation to CO_2_ emissions confirm that carbon emissions and drought are interconnected, with each influencing the other, indicating bidirectional causality. This two-way relationship supports the findings of Liu, Gudmundsson [51], who suggest that increased CO_2_ concentrations contribute to drought intensification, while droughts can also reduce carbon uptake in ecosystems [59]. This finding underscores the importance of addressing both CO_2_ emissions and water management to mitigate drought risk.

Also, the results presented that an increase in temperature raises the likelihood of drought in the countries. This finding is consistent with Trenberth, Dai [10], who argue that rising temperatures exacerbate drought conditions by increasing evaporation and water loss from soil and plants. However, the effect is weak, supporting the findings of Seneviratne, Corti [15], Dai, Zhao [50], Hirschi, Seneviratne [52], and Teuling, Van Loon [62], who also note the weak relationship between temperature and drought. The threshold level of temperature change required to mitigate drought effects is below a 1.30°C annual increase. This suggests that Iran and Saudi Arabia are particularly at risk of drought due to temperature increases. These countries should be aware that when the temperature increases by more than 1.30°C annually, drought is likely to occur. The causality test results in relation to temperature contrast with studies that emphasize the strong influence of temperature on drought occurrence, such as Teuling, Van Loon [62] and Dai, Zhao [50]. However, they support the study’s finding that the impact of temperature on drought is weak in comparison to the effects of rainfall and groundwater recharge.

In sum, the primary focus of this study is to investigate the magnitude of the impact of rainfall and groundwater recharge on mitigating drought. The findings show that both increased rainfall and groundwater recharge help mitigate drought. This aligns with the fact that drought is a prolonged period of deficient rainfall, leading to water scarcity, which negatively impacts agriculture, ecosystems, and economic stability [63]. Increasing rainfall and groundwater recharge replenishes aquifers, sustains surface water bodies, and supports ecological balance. Hydrological and climatological theories indicate that increased precipitation enhances groundwater infiltration and surface runoff, reducing drought severity [31]. Rainfall plays a crucial role in replenishing water sources, influencing both short-term and long-term hydrological cycles. Groundwater recharge is essential for maintaining hydrological equilibrium, especially in drought-prone areas. When ground water storage increases, it serves as a drought-resistant buffer, ensuring continued access to water during dry periods [64]. Moreover, enhanced groundwater recharge helps mitigate over-extraction, supporting long-term ecological balance.

## 6. Conclusion and policy recommendations

This study investigates the relationships between rainfall, groundwater recharge, and drought, using a global sample to identify strategies for mitigating drought. While drought is often considered a natural phenomenon, modern technologies have the potential to alter or influence its occurrence artificially. A nuanced understanding of these interrelated dynamics can offer crucial insights for enhancing drought resilience and fostering sustainable economic growth, ecosystem integrity, and environmental sustainability. Furthermore, CO_2_ emissions and temperature were included as key determinants in the analysis. The data spans from 1961 to 2022, and the techniques used in the analysis include the recent advancement of Diallo [6] to estimate Dynamic Panel Threshold Regression (DPThR) model and the Juodis, Karavias [7] Panel Non-causality Test. The study focuses on the top 10 countries with significant underground water overexploitation: India, China, Pakistan, Iran, Indonesia, Bangladesh, Saudi Arabia, Turkey, the USA, and Mexico, as identified in the UN World Water Development Report [33].

This study shows that hydro-climatic drivers overwhelmingly shape drought dynamics. Each additional millimeter of rainfall raises the SPEI by 0.003, lowering drought risk, with a mitigation threshold at 614.41 mm of annual rainfall; below this level, Pakistan, Iran, and the United States–Saudi Arabia region face heightened vulnerability. Groundwater recharge exerts an even stronger influence: a one-standard-deviation increase improves the SPEI by 5.06, and drought risk escalates when recharge drops below −0.0039 standard deviations, particularly in China, Iran, Mexico, Pakistan, Saudi Arabia, Turkey, and the United States. Temperature consistently worsens drought through intensified evapotranspiration and soil-moisture loss, while CO_2_ emissions show no direct, statistically significant effect within our specifications. Granger causality tests indicate a unidirectional flow from rainfall, groundwater recharge, temperature, and CO_2_ emissions to drought conditions, underscoring the primacy of hydro-climatic forces in driving drought variability. Theoretically, the findings of this study validate the hydroclimatic balance and environmental threshold theories, confirming that drought behavior exhibits a nonlinear and regime-dependent pattern. Practically, these results highlight the critical importance of maintaining rainfall adequacy and groundwater recharge above threshold levels, as this is essential for enhancing resilience to drought. The study emphasizes that sustaining these thresholds is key to mitigating drought risks, particularly in regions sensitive to climatic and hydrological changes.

Based on the findings, the following policy recommendations are introduced for global efforts to mitigate the effects of drought. Policymakers should prioritize managing annual rainfall, underground water and recharge, specifically considering the respective threshold values of these variables. For rainfall, authorities should implement measures to artificially manage rainfall when it is likely to fall below expected levels. This includes gathering and storing rainwater in tanks, reservoirs, or ponds for later use, using cloud seeding techniques with substances like salt or silver iodide to enhance precipitation, and employing ionization or ultrasound methods to stimulate rainfall. Furthermore, efforts to reduce runoff, increase infiltration, and protect watersheds should be prioritized, along with the use of efficient irrigation systems and schedules to maximize water use. Developing reservoirs, dams, and water storage facilities, as well as promoting conservation tillage, will help improve water retention. These measures, collectively, can reduce water loss and help manage rainfall more effectively, thus mitigating the consequences of drought. In the context of groundwater recharge, it is crucial to recognize that the effectiveness of recharge strategies is not only determined by precipitation levels but also by the interactions between surface water and groundwater, as well as the broader atmospheric conditions. These complex dynamics must be incorporated into water management policies to ensure sustainable drought mitigation and resilience, when authorities detect that recharge levels are falling below the threshold, steps should be taken to optimize recharge locations by selecting areas with high infiltration rates and proximity to aquifers. Efficient recharge methods, such as artificial recharge basins, recharge wells, and infiltration trenches, should be implemented, along with strategies to balance recharge with aquifer storage capacity and natural discharge rates. Managed aquifer recharge (MAR) should be used during wet periods to replenish aquifers with excess water, and aquifer storage and recovery (ASR) or groundwater banking should be employed to store water for future use. Moreover, it provides supporting evidence for the importance of integrating river–aquifer interactions, alongside the influence of both anthropogenic and climatic factors, in determining groundwater recharge efficiency and sustainability. These studies underscore that sustainable recharge strategies must consider not only natural hydrological processes but also human interventions and climatic variations, which collectively shape the long-term viability of groundwater resources. These policies, collectively, will help reduce the impacts of drought, enhance water management, and promote long-term environmental sustainability.

A limitation of the study is that it focuses solely on the top 10 countries with significant underground water overexploitation, which may not provide a comprehensive perspective on the broader phenomenon of drought. Additionally, the study does not consider factors such as topography or vegetation cover, which could also play important roles in drought dynamics. Future studies could address these aspects, depending on data availability. Furthermore, conducting a similar study on African Sub-Saharan countries could offer valuable insights into the relationship between drought and various other natural and human-related factors specific to that region. Moreover, additional research is required to gain a deeper understanding of the dynamic interactions between groundwater, surface water, and atmospheric processes. It is also essential to investigate the influence of both human and climatic drivers on these interconnected systems. Such research would provide valuable insights into how these factors collectively shape water availability and drought resilience, informing more effective and adaptive water resource management strategies.

## Supporting information

S1 Data Data. (XLSX)

## References

[1] Khansa H, Kawan S, Zanyar Othman O, Evan Fakhralddin M, Sulleyman S, Ali Mohammed S, et al. Addressing the issue of Poverty, Economic Growth, Health Matters, and Environmental Challenges. IJSRMST. 2025;4(3):18–32. doi: 10.59828/ijsrmst.v4i3.303

[2] Khan T, Samiullah M, Rouf I, Sultana S, Rahman S, Rahman B. The nexus of water scarcity and climate change: understanding interconnected challenges and formulating resilient strategies. Int J Environ Sci. 2024;7(3):57–68.

[3] UN-Water. Water Scarcity. UN-Water. 2023.

[4] Gebreslassie H, Berhane G, Gebreyohannes T, Hagos M, Hussien A, Walraevens K. Water Harvesting and Groundwater Recharge: A Comprehensive Review and Synthesis of Current Practices. Water. 2025;17(7):976. doi: 10.3390/w17070976

[5] UNESCO. The United Nations World Water Development Report 2022: Groundwater: Making the Invisible Visible. UNESCO. 2022.

[6] Diallo I. XTENDOTHRESDPD: Stata module to estimate a dynamic panel data threshold effects model with endogenous regressors. 2020.

[7] Juodis A, Karavias Y, Sarafidis V. A homogeneous approach to testing for Granger non-causality in heterogeneous panels. Empir Econ. 2020;60(1):93–112. doi: 10.1007/s00181-020-01970-9

[8] NASA. Warming Makes Droughts, Extreme Wet Events More Frequent, Intense. https://www.nasa.gov/centers-and-facilities/goddard/warming-makes-droughts-extreme-wet-events-more-frequent-intense/. 2023.

[9] Masson-Delmotte V, Zhai P, Pirani A, Connors SL, Péan C, Berger S, et al. Climate change 2021: the physical science basis. Contribution of working group I to the sixth assessment report of the intergovernmental panel on climate change. 2021;2(1):2391.

[10] Trenberth KE, Dai A, van der Schrier G, Jones PD, Barichivich J, Briffa KR, et al. Global warming and changes in drought. Nature Clim Change. 2013;4(1):17–22. doi: 10.1038/nclimate2067

[11] Wilhite DA. Drought as a natural hazard: concepts and definitions. Droughts. Routledge. 2016:3–18.

[12] Hayes M, Svoboda M, Wall N, Widhalm M. The Lincoln Declaration on Drought Indices: Universal Meteorological Drought Index Recommended. Bulletin of the American Meteorological Society. 2011;92(4):485–8. doi: 10.1175/2010bams3103.1

[13] Karl TR, Trenberth KE. Modern global climate change. Science. 2003;302(5651):1719–23. doi: 10.1126/science.1090228 14657489

[14] Van Loon AF. Hydrological drought explained. WIREs Water. 2015;2(4):359–92. doi: 10.1002/wat2.1085

[15] Seneviratne SI, Corti T, Davin EL, Hirschi M, Jaeger EB, Lehner I, et al. Investigating soil moisture–climate interactions in a changing climate: A review. Earth-Science Reviews. 2010;99(3–4):125–61. doi: 10.1016/j.earscirev.2010.02.004

[16] Wilhite DA. Drought policy and preparedness: the Australian experience in an international context. From disaster response to risk management: Australia’s national drought policy. Springer. 2005:157–76.

[17] Hisdal H, Stahl K, Tallaksen LM, Demuth S. Have streamflow droughts in Europe become more severe or frequent? Intl Journal of Climatology. 2001;21(3):317–33. doi: 10.1002/joc.619

[18] Gocic M, Trajkovic S. Analysis of changes in meteorological variables using Mann-Kendall and Sen’s slope estimator statistical tests in Serbia. Global and Planetary Change. 2013;100:172–82. doi: 10.1016/j.gloplacha.2012.10.014

[19] Ogunrinde AT, Oguntunde PG, Akinwumiju AS, Fasinmirin JT. Analysis of recent changes in rainfall and drought indices in Nigeria, 1981–2015. Hydrological Sciences Journal. 2019;64(14):1755–68. doi: 10.1080/02626667.2019.1673396

[20] Barthel R, Stangefelt M, Giese M, Nygren M, Seftigen K, Chen D. Current understanding of groundwater recharge and groundwater drought in Sweden compared to countries with similar geology and climate. Geografiska Annaler: Series A, Physical Geography. 2021;103(4):323–45. doi: 10.1080/04353676.2021.1969130

[21] Sheffield J, Wood EF, Roderick ML. Little change in global drought over the past 60 years. Nature. 2012;491(7424):435–8. doi: 10.1038/nature11575 23151587

[22] Dai A. Drought under global warming: a review. WIREs Climate Change. 2010;2(1):45–65. doi: 10.1002/wcc.81

[23] Gutzler DS, Robbins TO. Climate variability and projected change in the western United States: regional downscaling and drought statistics. Clim Dyn. 2010;37(5–6):835–49. doi: 10.1007/s00382-010-0838-7

[24] USGS. Groundwater Recharge. U.S. Geological Survey. 2023. https://www.usgs.gov/water-science-school

[25] Field CB, Barros VR. Climate change 2014–impacts, adaptation and vulnerability: regional aspects. Cambridge University Press. 2014.

[26] Arnell N. Climate change and global water resources. Global Environmental Change. 1999;9:S31–49. doi: 10.1016/s0959-3780(99)00017-5

[27] Hiscock KM, Rivett MO, Davison RM. Sustainable groundwater development. 2002.

[28] Sophocleous M. Interactions between groundwater and surface water: the state of the science. Hydrogeology Journal. 2002;10(2):348–348. doi: 10.1007/s10040-002-0204-x

[29] McEvoy A, Famiglietti JS, Liu PW, Reager JT. From drought to recovery: a GRACE-based assessment of groundwater storage variations in California. In: AGU Fall Meeting Abstracts, 2017.

[30] Guo Z, Dirmeyer PA, Koster RD, Sud Y, Bonan G, Oleson KW. GLACE: the global land–atmosphere coupling experiment. Part II: analysis. Journal of Hydrometeorology. 2006;7(4):611–25.

[31] Scanlon BR, Keese KE, Flint AL, Flint LE, Gaye CB, Edmunds WM, et al. Global synthesis of groundwater recharge in semiarid and arid regions. Hydrological Processes. 2006;20(15):3335–70. doi: 10.1002/hyp.6335

[32] Global SPEI database (SPEIbase). 2023.

[33] UNWWDR. The United Nations World Water Development Report 2022: Groundwater: making the invisible visible. 2022.

[34] WDI. 2023.

[35] CCKP. 2023.

[36] India Environment Portal. https://www.indiaenvironmentportal.org.in/. 2023.

[37] GAP. Gapminder. https://www.gapminder.org. 2023.

[38] Andualem TG, Demeke GG, Ahmed I, Dar MA, Yibeltal M. Groundwater recharge estimation using empirical methods from rainfall and streamflow records. Journal of Hydrology: Regional Studies. 2021;37:100917. doi: 10.1016/j.ejrh.2021.100917

[39] Addisie MB. Groundwater recharge estimation using water table fluctuation and empirical methods. H2Open Journal. 2022;5(3):457–68. doi: 10.2166/h2oj.2022.026

[40] Chaturvedi R. A note on the investigation of ground water resources in western districts of Uttar Pradesh. 1973.

[41] Kumar C, Seethapathi P. Assessment of natural groundwater recharge in Upper Ganga Canal command area. Journal of Applied Hydrology. 2002;15(4):13–20.

[42] Ali M, Mubarak S, Islam A, Biswas P. Comparative evaluation of various empirical methods for estimating groundwater recharge. Arch Curr Res Int. 2017;11(1):1–10.

[43] PR KR. Hydrometeorological aspects of estimating groundwater potential. 1970.

[44] Kirchner JOG, Van Tonder GJ, Lukas E. Exploitation potential of Karoo aquifers. Water Research Commission. 1991.

[45] Bredenkamp D, Botha L, Van Tonder G, Van Rensburg HJ. Manual on quantitative estimation of groundwater recharge and aquifer storativity: based on practical hydro-logical methods. Water Research Commission. 1995.

[46] Deshbhandari PG, Krishnaiah C. Comparative analysis of empirical models derived groundwater recharge estimation in Venkatapura watershed, Karnataka. 2017. https://rsisinternational.org/IJRSI/Issue42/07-10.pdf

[47] Maxey GB, Eakin TE. Ground water in White River Valley, White Pine, Nye, and Lincoln Counties, Nevada. 1949.

[48] Seo MH, Kim S, Kim Y-J. Estimation of dynamic panel threshold model using Stata. The Stata Journal: Promoting communications on statistics and Stata. 2019;19(3):685–97. doi: 10.1177/1536867x19874243

[49] Wilhite DA, Sivakumar MVK, Pulwarty R. Managing drought risk in a changing climate: The role of national drought policy. Weather and Climate Extremes. 2014;3:4–13. doi: 10.1016/j.wace.2014.01.002

[50] Dai A, Zhao T, Chen J. Climate Change and Drought: a Precipitation and Evaporation Perspective. Curr Clim Change Rep. 2018;4(3):301–12. doi: 10.1007/s40641-018-0101-6

[51] Liu L, Gudmundsson L, Hauser M, Qin D, Li S, Seneviratne SI. Revisiting assessments of ecosystem drought recovery. Environ Res Lett. 2019;14(11):114028. doi: 10.1088/1748-9326/ab4c61

[52] Hirschi M, Seneviratne SI, Alexandrov V, Boberg F, Boroneant C, Christensen OB, et al. Observational evidence for soil-moisture impact on hot extremes in southeastern Europe. Nature Geosci. 2010;4(1):17–21. doi: 10.1038/ngeo1032

[53] Berg A, Lintner B, Findell K, Giannini A. Soil Moisture Influence on Seasonality and Large-Scale Circulation in Simulations of the West African Monsoon. J Climate. 2017;30(7):2295–317. doi: 10.1175/jcli-d-15-0877.1

[54] Adib A, Moradi A, Lotfirad M, Azizipour M, Liaghat A. Investigating the relationship between meteorological, hydrological and groundwater resource droughts under the influence of upstream dam reservoir effects. Model Earth Syst Environ. 2023;9(3):3609–19. doi: 10.1007/s40808-023-01710-9

[55] Henao Casas JD, Fernández Escalante E, Ayuga F. Alleviating drought and water scarcity in the Mediterranean region through managed aquifer recharge. Hydrogeol J. 2022;30(6):1685–99. doi: 10.1007/s10040-022-02513-5

[56] Zaerpour M, Hatami S, Ballarin AS, Papalexiou SM, Pietroniro A, Adamowski JF. Climate shapes baseflows, influencing drought severity. Environ Res Lett. 2024;20(1):014035. doi: 10.1088/1748-9326/ad975a

[57] Jasechko S, Seybold H, Perrone D, Fan Y, Kirchner JW. Widespread potential loss of streamflow into underlying aquifers across the USA. Nature. 2021;591(7850):391–5. doi: 10.1038/s41586-021-03311-x 33731949

[58] Reichstein M, Bahn M, Ciais P, Frank D, Mahecha MD, Seneviratne SI, et al. Climate extremes and the carbon cycle. Nature. 2013;500(7462):287–95. doi: 10.1038/nature12350 23955228

[59] Schwalm CR, Williams CA, Schaefer K, Baldocchi D, Black TA, Goldstein AH, et al. Reduction in carbon uptake during turn of the century drought in western North America. Nature Geosci. 2012;5(8):551–6. doi: 10.1038/ngeo1529

[60] Bowman DMJS, Williamson GJ, Abatzoglou JT, Kolden CA, Cochrane MA, Smith AMS. Human exposure and sensitivity to globally extreme wildfire events. Nat Ecol Evol. 2017;1(3):58. doi: 10.1038/s41559-016-0058 28812737

[61] Houghton RA, House JI, Pongratz J, van der Werf GR, DeFries RS, Hansen MC, et al. Carbon emissions from land use and land-cover change. Biogeosciences. 2012;9(12):5125–42. doi: 10.5194/bg-9-5125-2012

[62] Teuling AJ, Van Loon AF, Seneviratne SI, Lehner I, Aubinet M, Heinesch B, et al. Evapotranspiration amplifies European summer drought. Geophysical Research Letters. 2013;40(10):2071–5. doi: 10.1002/grl.50495

[63] Wilhite DA, Glantz MH. Understanding: the Drought Phenomenon: The Role of Definitions. Water International. 1985;10(3):111–20. doi: 10.1080/02508068508686328

[64] Rodell M, Velicogna I, Famiglietti JS. Satellite-based estimates of groundwater depletion in India. Nature. 2009;460(7258):999–1002. doi: 10.1038/nature08238 19675570

